# Individual-level associations between implementation leadership, climate, and anticipated outcomes: a time-lagged mediation analysis

**DOI:** 10.1186/s43058-023-00459-7

**Published:** 2023-07-11

**Authors:** Karina Myhren Egeland, Randi Hovden Borge, Nadina Peters, Harald Bækkelund, Nora Braathu, Marisa Sklar, Gregory A. Aarons, Ane-Marthe Solheim Skar

**Affiliations:** 1 Norwegian Centre for Violence and Traumatic Stress Studies (NKVTS), Gullhaugveien 1, 0484 Oslo, Norway; 2 National Institute of Occupational Health, Gydas vei 8, 0363 Oslo, Norway; 3 Department of Psychiatry, University of California, San Diego, 9500 Gilman Drive (0812), La Jolla, CA 92093-0812 USA; 4 Diego ACTRI Dissemination and Implementation Science Center, UC San, 9500 Gilman Drive, La Jolla, CA 92093 USA; 5 Child and Adolescent Services Research Center, 3665 Kearny Villa Rd., Suite 200N, San Diego, CA 92123 USA

**Keywords:** Implementation climate, Feasibility, Appropriateness, Acceptability, Implementation leadership

## Abstract

**Background:**

Leaders can improve implementation outcomes by developing an organizational climate conducive to the implementation of evidence-based practices (EBP). This study tested the lagged associations between individual-level perceptions of implementation leadership, implementation climate, and three anticipated implementation outcomes, that is EBP acceptability, appropriateness, and feasibility.

**Methods:**

Screening tools and treatment methods for posttraumatic stress disorder were implemented in 43 Norwegian mental health services. A sample of 494 child and adult mental health care professionals (*M* = 43 years, 78% female) completed surveys addressing perceptions of first-level leaders’ (*n* = 47) implementation leadership and their clinics’ implementation climate. Single-level structural equation models estimating both direct, indirect, and total effects were used to investigate whether perceived implementation climate mediated the association between perceived implementation leadership and perceived acceptability, appropriateness, and feasibility of screening tools and treatment methods.

**Results:**

Regarding the treatment methods, implementation leadership was associated with therapists’ perceptions of acceptability, appropriateness, and feasibility. Implementation climate also mediated between implementation leadership and the outcomes. Regarding the screening tools, implementation leadership was not associated with the outcomes. However, implementation climate mediated between implementation leadership and therapists’ perceptions of acceptability and feasibility, but not appropriateness. Analyses with the implementation climate subscales showed stronger associations for therapists’ perceptions of the treatment methods than of screening tools.

**Conclusions:**

Leaders may promote positive implementation outcomes, both directly and through implementation climate. With regard to the effect sizes and explained variance, results indicated that both implementation leadership and implementation climate were more strongly associated with the therapists’ perceptions of the treatment methods, implemented by one group of therapists, than the screening tools, implemented by all therapists. This may imply that implementation leadership and climate may have stronger effects for smaller implementation teams within a larger system than for system-wide implementations or when the clinical interventions being implemented are more complex rather than simple ones.

**Trial registration:**

ClinicalTrials NCT03719651, 25 October 2018.

Contributions to the literature

- Understanding the association between implementation leadership and climate, and implementation outcomes, can give insight into the importance of these factors for succeeding with the implementation of evidence-based practices.
- The findings showed that perceived implementation climate mediated the associations between implementation leadership and the three implementation outcomes measured: therapists’ perceptions of acceptability, appropriateness, and feasibility of evidence-based practices.
- This study contributes novel information related to how leaders can affect implementation outcomes both directly and through a positive implementation climate.

## Background

To improve care in mental health services, a major goal is to increase the use of evidence-based practices (EBPs). Although much attention has been paid to strategies that can lead to successful implementation [1], we have a poor understanding of how and why different implementation strategies work. That is, to understand the mechanisms that affect implementation outcomes [2–4]. More recent attention has focused on the concepts of perceived implementation leadership and implementation climate, which are believed to be important for successful EBP implementation in health services [5–7]. Aarons and colleagues [7] suggest in their theory that implementation leadership will improve implementation outcomes by creating a positive strategic organizational climate to support EBP implementation. In this study, we investigate whether perceived EBP implementation climate mediates the associations between perceived implementation leadership and mental health practitioners’ perceptions of EBP acceptability, appropriateness, and feasibility.

### Leading through a strategic climate

Health care leaders, and especially first-level leaders (leaders who supervise and manage frontline employees), are believed to have an important, yet poorly understood role in the implementation of EBPs [8, 9]. There are several implementation theories offering explanations for how leaders can positively influence EBP implementation. Some theories emphasize the influence of leaders’ commitment to EBP implementation on implementation effectiveness [8]. Other theories emphasize behaviors leaders can take to develop a strategic climate for implementation by being supportive, knowledgeable, proactive, and perseverant about implementing specific EBPs [10]. While some differences exist between these leadership implementation theories, they do converge on the theoretical proposition that leaders can serve as change agents in their organizations to promote implementation effectiveness.

Implementation outcomes are defined as the effects of deliberate and purposive actions to implement new practices [11, 12] and can be divided into anticipated outcomes (i.e., the likelihood the practice will be adopted or delivered) and actual outcomes (i.e., the extent the practice is adopted or delivered) [13]. Anticipated outcomes, such as practitioners’ perceptions of EBP acceptability, appropriateness, and feasibility [12], are thought to be precursors and/or determinants of actual outcomes. Findings of direct relationships between implementation leadership and implementation outcomes are sparse and inconsistent [14]. Of two studies that have examined the relationship between implementation leadership and adoption of EBPs, one study found a positive relationship [15] whereas the other did not [16].

Organization theory posits that implementation climate is assumed to operate as a mechanism between implementation leadership and implementation outcomes [7]. This is consistent with theories of climate as a mediator of outcomes in the broader organizational literature [17]. Implementation climate is a type of strategic climate defined as employees’ shared perceptions of whether the specific EBP is expected, rewarded, and supported by the organization [18, 19]. A positive implementation climate means that employees clearly understand and experience that leaders support and facilitate the use of the practice [6]. There are several policies, practices, and procedures through which employees may perceive the importance of EBP implementation in their organization. Based on the strategic climate literature, relevant implementation climate dimensions thought to impact the implementation of EBPs are as follows: (1) whether the organization recruits and selects employees that are open for change, (2) whether staff are recognized for their EBP use, (3) whether staff are recruited and selected based on their experience with EBP, (4) whether the organization focuses on EBP use, (5) whether the organization provides EBP education and training opportunities, and (6) whether the organization rewards EBP use [18].

Organizational climate has been proposed as both an individual-level construct, referring to individuals’ own perceptions of a psychological organizational climate, and an organizational-level construct, referring to employees’ agreed perceptions of the climate [20]. Implementation climate at the unit-level is often favored [5, 21]. Some have argued that to fully understand the importance of organizational climate and how it relates to other constructs, it would be preferrable to examine both individual- and unit-level climate [22, 23]. Focusing on the individual level, organizational climate can be construed as a personal/individual level factor rather than a situational or unit level factor. From leaders’ perspective, who are often those who shape the climate [7], it can be expedient to aim their leadership initiatives both at the individual and the organizational level [22]. In this study, we examined how individual leaders’ behaviors relates to individual therapists’ perceptions of acceptability, appropriateness, and feasibility of interventions, both directly and through therapists’ perceived implementation climate. To test this, implementation climate was measured at the individual level.

Although the literature is still sparse, some cross-sectional [24–26] and longitudinal [15, 27] studies have shown that first-level leaders’ implementation leadership are linked to EBP implementation climate. There is also growing evidence supporting that implementation climate is related to implementation outcomes such as self-reported EBP use [15, 28–30] and fidelity to EBP [27, 31], but these relationships were also affected by third-variables such as molar organizational climate [28] and complexity of the practice being implemented or utilized [27].

To understand whether implementation climate operates as a mechanism between implementation leadership and implementation outcomes, mediation analyses with prospective or longitudinal designs may be used [3, 32]. Two longitudinal studies have examined implementation climate as a mediator between implementation leadership and implementation outcomes [15, 27]. Williams and colleagues’ [15] study included 30 outpatient children’s mental health clinics and showed that improvements in implementation leadership significantly increased the level of EBP implementation climate, which then significantly increased average employee-reported EBP use. In a similar study including 65 schools, Williams and colleagues [27] showed that principals’ increased implementation leadership predicted higher EBP implementation climate, which in turn predicted higher fidelity to school-based EBPs for autism.

Due to sparse empirical evidence currently supporting the theoretical assumption of a leadership-climate-outcome relationship, studies using prospective designs are needed [33, 34]. An empirical rigorous approach is to examine associations between variables measured at different time points (i.e., lagged associations). In this study, the Leadership and Organizational Change for Implementation (LOCI) strategy was administered to leaders in the participating clinics [35]. LOCI is a 12-month implementation strategy that aims to improve implementation leadership in order to create a positive strategic organizational climate that supports EBP implementation [7]. Accordingly, we wanted to investigate if perceived implementation leadership measured at the time the leaders received the LOCI strategy (T1) was associated with perceived implementation climate 4 months later (T2) and perceptions of the screening tools and treatment methods at the end of the implementation period (T3). The following hypotheses were examined:

- H1: Implementation leadership (T1) is associated with EBP acceptability, appropriateness, and feasibility 8 months later (T3).
- H2: Implementation leadership (T1) is associated with perceived implementation climate 4 months later (T2).
- H3: Perceived implementation climate (T2) is associated with EBP acceptability, appropriateness, and feasibility 4 months later (T3).
- H4: Perceived implementation climate at T2 mediates the association between implementation leadership at T1 and EBP acceptability, appropriateness, and feasibility at T3.

As we know little about how implementation climate affects implementation outcomes, we also investigated whether and how the subdimensions of perceived implementation climate mediated between implementation leadership and EBP acceptability, appropriateness, and feasibility. Since few have previously examined these relationships, exploratory analyses were conducted based on an expectation that the subdimensions might differ in how they relate to both implementation leadership and implementation outcomes, which in turn might produce differences in mediation.

## Methods

### Setting

This study was part of a national implementation of both *screening tools* for exposure to potentially traumatizing events and related posttraumatic stress symptoms and evidence-based *treatment methods* for posttraumatic stress disorder (PTSD) in Norwegian child and adult specialized mental health clinics [35].

### Participants

The study sample included 494 therapists with an average age of 43 years, of whom most were female (Table 1), clinical psychologists, and psychiatrists. Therapists completed surveys addressing their perceptions of their clinics’ (*n* = 43) implementation climate and their first-level leaders’ implementation leadership. The leaders’ (*n* = 47) average age was 50 years. Approximately half were clinical psychologists, and the rest were clinical social workers, psychiatric nurses, and psychiatrists.

**Table 1:** Participant demographics (*N* = 494)

**Gender**	
Women	323 (78.40%)
Men	89 (21.60%)
(missing)	82
**Age**	
Mean (SD)	43.32 (10.86)
(missing)	82
**Work experience (years)**	
Mean (SD)	11.78 (9.51)
(missing)	132
**Tenure in organization (years)**	
Mean (SD)	5.71 (6.78)
(missing)	160
**Trained in EBPs for PTSD**	
Screening tools only	310 (62.75%)
Screening tools and treatment methods	184 (37.24%)

### Implementation strategy

The implementation took place between August 2018 and May 2020. First, therapists in all clinics were trained in screening of trauma exposure and posttraumatic stress symptoms and diagnosing of PTSD. Second, a subgroup of therapists received training and supervision in either Trauma-Focused Cognitive Behavioral Therapy (TF-CBT) (child clinics) or Eye Movement Desensitization and Reprocessing (EMDR) or Cognitive Therapy for PTSD (CT-PTSD) (adult clinics), three of the most well-documented EBPs for PTSD [36, 37].

To support the implementation, the clinics were randomly allocated into one of three cohorts that received the 12-months’ LOCI strategy in the active implementation phase [38] on overlapping time points (see Table 2). During LOCI, first-level leaders (i.e., those who directly supervise therapists) participated in 5 days of training (2 days of start-up, booster session after 4 and 8 months, and graduation after 12 months) spread over 1 year. They received feedback reports based on 360° assessments on their leadership and the clinic’s implementation climate and co-developed individualized leadership development plans with LOCI trainers/coaches. First-level leaders also participated in weekly coaching calls, and organizational strategy meetings to inform the development of an organizational climate development plan (see [39] for a detailed description of LOCI).

**Table 2:** Implementation and survey plan

**Cohort**	**Time period**						
					**T1**^**a**^	**T2**^**a**^	**T3**^**a**^
	September 2018	September 2018	January 2019	May 2019	September 2019	January 2020	May 2020
I	EBP training	LOCI start-up	LOCI booster	LOCI booster	LOCI graduation	Sustainment phase	Sustainment phase
II	EBP training		LOCI start-up	LOCI booster	LOCI booster	LOCI graduation	Sustainment phase
III	EBP training			LOCI start-up	LOCI booster	LOCI booster	LOCI graduation

^a^T1 survey: August 2019; T2 survey: December 2019; T3 survey: April 2020

### Procedure

Therapists completed surveys addressing their perceptions of first-level leaders’ implementation leadership (T1; August 2019), the clinics’ implementation climate (T2; December 2019), and whether they found the screening tools and treatment methods acceptable, appropriate, and feasible (T3; April 2020) (Table 2). The first survey (T1, implementation leadership, *N* = 360) was completed when leaders from all cohorts had participated in LOCI for a minimum of 3 months. The second survey (T2; implementation climate, *N* = 304) was completed when cohort I was graduating from LOCI and entering the sustainment phase. Cohorts II and III were still participating in LOCI. The third survey (T3; implementation outcomes, *N* = 254) was completed when cohort II was graduating from LOCI and entering their sustainment phase. Cohort III was still actively participating in LOCI. Across time points, 216 therapists had responded to both T1 and T2 surveys, 176 to both T1 and T3 surveys, and 171 to both T2 and T3 surveys. In sum, 139 therapists had responded to all three time points.

### Measures

*The implementation leadership scale (ILS)* consists of 12 items measuring leadership behavior thought to impact the EBP implementation [10] across four subdimensions. The therapists were asked to evaluate their first-level leaders’ proactive, knowledgeable, supportive, and perseverant leadership towards the implementation of EBPs for PTSD on a scale between 0 (not at all) to 4 (to a very great extent). A higher score indicates more favorable implementation leadership. ILS demonstrated excellent internal consistency (*α* = 0.97), in addition to good psychometric properties in the main study [40].

*The implementation climate scale (ICS)* consists of 18 items measuring global and six subdimensions of implementation climate [18]. The therapists were asked to evaluate their clinic’s: (1) Focus on EBP, (2) Educational support for EBP, (3) Recognition for EBP, (4) Rewards for EBP, (5) Selection for EBP, and (6) Selection for openness on a scale between 0 (not at all) to 4 (to a very great extent). The questions were directed towards EBPs for PTSD, specifically. Items are scored on a scale between 0 (not at all) to 4 (to a very great extent). A higher score indicates more favorable implementation climate. The scale showed excellent internal consistency reliability in the current study [41]. However, due to psychometric challenges as a result of financial rewards/incentives and promotions being rare in Norwegian working conditions [41], the Rewards subscale and item 9 from the Recognition subscale (more likely to be promoted) were excluded from the ICS global scale (hereby called ICS-r), resulting in 14 items.

In the subscales analyses, the Rewards subscale was included. Item 9 was excluded from the Recognition subscale, hereby called the Recognition-r subscale. Recognition-r showed excellent internal reliability (*α* = 0.90).

*EBP acceptability, appropriateness, and feasibility of screening tools and EBP treatment methods* were measured using the Acceptability of Intervention Measure (AIM), Intervention Appropriateness Measure (IAM), and Feasibility of Intervention Measure (FIM), respectively [12]. These measures were developed to measure three implementation outcomes, as indicated by Proctor and colleagues [11]. The therapists were asked to evaluate separately whether they experienced (a) the PTSD screening tools and (b) the PTSD treatment methods as acceptable, appropriate, and feasible in their clinic. Items were scored from 1 (completely disagree) to 5 (completely agree). A higher score indicates more favorable acceptability, appropriateness, and feasibility. To account for potential measurement error due to few indicators, the outcome measures were included in the model as latent variables. Standardized factor loadings are shown in Table 3. All loadings were above 0.6 and statistically significant.

**Table 3:** Standardized factor loadings for the latent outcome variables

**Item**	**Standardized** **factor loadings**
**Perceptions of trauma screening tools**	
Acceptability	
Item 1	0.69
Item 2	0.94
Item 3	0.95
Item 4	0.87
Appropriateness	
Item 5	0.93
Item 6	0.98
Item 7	0.96
Item 8	0.96
Feasibility	
Item 9	0.92
Item 10	0.96
Item 11	0.97
Item 12	0.89
**Perceptions of trauma treatment methods**	
Acceptability	
Item 1	0.80
Item 2	0.91
Item 3	0.95
Item 4	0.94
Appropriateness	
Item 5	0.85
Item 6	0.91
Item 7	0.93
Item 8	0.90
Feasibility	
Item 9	0.95
Item 10	0.92
Item 11	0.93
Item 12	0.78

All loadings significant at *p* < 0.001

*Control variables* in all models included LOCI cohort, age, sex, years of work experience, and organizational tenure. LOCI cohort was used as a control variable for both the leadership and the implementation outcomes and the implementation climate and the outcomes. As for age, years of work experience, and tenure, these may be indicative of the employees’ professional experience and managerial skills. Sex is included as women make up the majority of the mental health services workforce.

### Statistical analyses

**Fig. 1:**
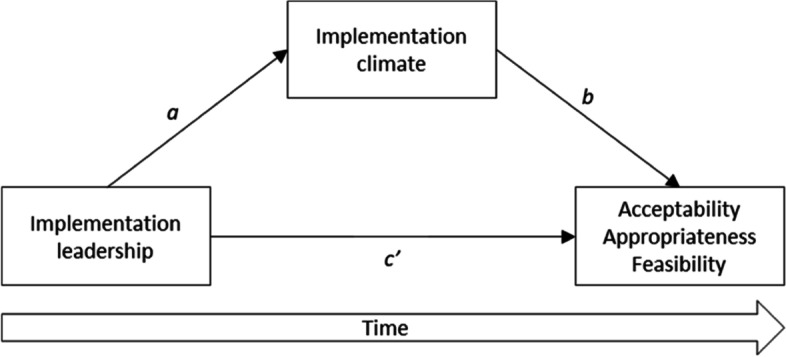
Conceptual model of relationships between implementation leadership, implementation climate, and anticipated implementation outcomes

Study hypotheses were investigated with structural equation modeling (SEM) in Mplus 8.3 [42] adjusting for the nested data structure (TYPE=COMPLEX) using full information maximum likelihood estimation with robust standard errors (MLR). Measurement error may lead to both over- and underestimation of path coefficients in models investigating mediation [43]. As potential measurement error can be considerable when the number of indicators is low, we used latent variable modeling for the measures with few indicators (i.e., AIM, IAM, and FIM, and the ICS subscales). The ILS (12 items) and the ICS-r (14 items) both have many indicators and were included as manifest variables based on mean scores calculated by averaging across items to reduce model complexity. Since aggregation indices were below recommended thresholds for aggregating data, implementation climate was analyzed at the individual level [44]. We estimated a series of mediation models with direct, indirect (via perceived implementation climate), and total effects of implementation leadership on therapists’ perceptions of EBP acceptability, appropriateness, and feasibility (Fig. 1). Specifically, each outcome measure at T3 was regressed on implementation leadership at T1 (path *c’*, i.e., *direct* path) and perceived implementation climate at T2 (path *b*). Perceived implementation climate at T2 was also regressed on implementation leadership at T1 (path *a*). The indirect effect of implementation leadership was calculated as the product of paths *a* and *b* (*indirect* = *a* × *b*). We ran separate models for each of the three outcome measures, for each intervention (i.e., screening tools, treatment methods), and each mediator variable (i.e., perceived global and subscale implementation climate), resulting in six models for each mediator variable. Since EBP acceptability, appropriateness, and feasibility were only measured at the end of the implementation period, we were not able to control for baseline levels in the outcome variables. In models predicting perceptions of treatment methods, only therapists who were trained in one of the treatment methods were included in the analyses.

Distributions of indirect effects are known to violate the normality assumption on which frequentist methods are based. Additionally, upon inspection, distributions of all our outcome variables were skewed. Thus, a bootstrapping procedure—repeatedly drawing 1000 samples from the original sample to estimate sampling distributions—was used instead of traditional hypothesis testing. To test study hypotheses, we utilized 95% confidence intervals (CIs) based on 2.5. and 97.5 percentile values of the sampling distributions for each parameter. Parameters were considered significantly different from zero when their 95% CIs did not cross zero.

## Results

On average, the therapists rated implementation leadership and perceived implementation climate at 2.51 and 2.37, respectively. Acceptability, appropriateness, and feasibility scores for both screening tools and treatment methods were generally very high (Table 4).

**Table 4:** Descriptive statistics for the study variables

	***N***	***M***	***SD***
**Implementation leadership** (range 0–4)	360	2.51	0.89
**Implementation climate revised (ICS-r)** (range 0–4)	304	2.37	0.66
Focus		2.74	0.85
Education support		2.16	0.98
Recognition-r		2.60	0.91
Rewards		0.73	0.74
Selection for EBP		1.72	0.91
Selection for openness		2.71	0.91
**Perceptions of screening tools** (range 1–5)	254		
Acceptability of screening tools		4.19	0.74
Appropriateness of screening tools		4.16	0.77
Feasibility of screening tools		4.31	0.72
**Perceptions of treatment methods** (range 1–5)	131		
Acceptability of treatment methods		4.44	0.60
Appropriateness of treatment methods		4.26	0.68
Feasibility of treatment methods		4.13	0.75

Means and SDs for all variables are based on mean scores. Implementation climate revised is a revised version of the ICS consisting of 14 items (excluding item 9 and the Rewards subscale). Recognition-r does not include item 9 from the original ICS

### Associations between implementation leadership and implementation outcomes

Results from the main analyses with implementation climate as mediator are displayed in Table 5. Total effects of implementation leadership on acceptability, appropriateness, and feasibility only partially supported Hypothesis 1 that implementation leadership assessed at T1 would be associated with EBP acceptability, appropriateness, and feasibility assessed 8 months later (*total* parameters). Specifically, all estimates were positive but statistically significant only for acceptability, appropriateness, and feasibility of the *treatment methods.*

**Table 5:** Parameter estimates for direct effects, indirect effects (through implementation climate), and total effects of implementation leadership on anticipated outcomes of screening tools and treatment methods at T3

Outcome	Parameter	**Screening tools (*N* = 494)**		**Treatment methods (*N* = 184)**	
		Estimate (s.e.)	95% CI	Estimate (s.e.)	95% CI
Implementation climate	*a*	**0.44 (0.05)**	0.35, 0.55	**0.40 (0.08)**	0.27, 0.56
Acceptability	*b*	**0.21(0.10)**	0.05, 0.43	**0.24 (0.10)**	0.05, 0.42
	*direct*	− 0.02 (0.07)	− 0.16, 0.10	0.10 (0.06)	− 0.03, 0.23
	*indirect*	**0.09 (0.05)**	0.02, 0.19	**0.09 (0.04)**	0.02, 0.18
	*total*	0.07 (0.05)	− 0.03, 0.17	**0.19 (0.05)**	0.09, 0.30
	*R*^*2*^	0.07		0.23	
Appropriateness	*b*	0.17 (0.11)	− 0.02, 0.41	**0.25 (0.13)**	0.01, 0.51
	*direct*	0.05 (0.10)	− 0.16, 0.23	0.15 (0.08)	− 0.02, 0.31
	*indirect*	0.08 (0.05)	− 0.01, 0.19	**0.10 (0.05)**	0.01, 0.22
	*total*	0.13 (0.08)	− 0.04, 0.28	**0.25 (0.08)**	0.10, 0.39
	*R*^*2*^	0.06		0.17	
Feasibility	*b*	**0.21 (0.10)**	0.02, 0.43	**0.40 (0.15)**	0.11, 0.70
	*direct*	− 0.03 (0.10)	− 0.21, 0.16	0.15 (0.11)	− 0.08, 0.35
	*indirect*	**0.09(0.05)**	0.01, 0.19	**0.16 (0.07)**	0.04, 0.32
	*total*	0.06 (0.08)	− 0.08, 0.22	**0.31 (0.09)**	0.12, 0.48
	*R*^*2*^	0.07		0.21	

Implementation climate is a revised version of the ICS consisting of 14 items (excluding item 9 and the Rewards subscale). 95% bootstrapped confidence intervals (CIs) for all parameters. Parameters in bold have CIs that do not cross zero. Cohort, age, gender, years of work experience in current occupation, and organizational tenure controlled for in all models

### Associations between implementation leadership and perceived implementation climate

The results indicated significant positive associations between implementation leadership and implementation climate among all therapists and in the subsample of therapists trained in the *treatment methods* (Table 5, *a* parameters). The results also indicated significant positive associations between implementation leadership and all subscales in the ICS, except the Rewards subscale where the estimates were close to zero and non-significant (Tables 6 and 7, *a* parameters). Thus, the results supported hypothesis 2 that implementation leadership assessed at T1 would be associated with perceived implementation climate assessed 4 months later (T2).

**Table 6:** Parameter estimates for direct and indirect effects (through implementation climate subscales) of implementation leadership on anticipated outcomes of screening tools at T3 (*N* = 494)

Outcome	Parameter	Mediator					
		Focus	Education support	Recognition-r	Rewards	Selection for EBP	Selection for openness
Implementation outcome							
	*a*	**0.52 (0.07)** [0.39, 0.66]	**0.53 (0.08)** [0.39, 0.71]	**0.27 (0.09)** [0.12, 0.46]	0.04 (0.07) [− 0.07, 0.20]	**0.36 (0.07)** [0.23, 0.48]	**0.43 (0.07)** [0.28, 0.58]
Acceptability (AIM)							
	*b*	0.11 (0.08) [− 0.03, 0.26]	0.05 (0.06) [− 0.05, 0.18]	**0.15 (0.07)** [0.03, 0.29]	− 0.23 (0.10) [− 0.41, 0.02]	0.07 (0.07) [− 0.05, 0.21]	0.07 (0.07) [− 0.07, 0.21]
	*direct*	0.02 (0.07) [− 0.13, 0.15]	0.06 (0.07) [− 0.08, 0.17]	0.05 (0.06) [− 0.07, 0.15]	0.09 (0.06) [− 0.02, 0.20]	0.07 (0.06) [− 0.07, 0.17]	0.05 (0.06) [− 0.08, 0.16]
	*indirect*	0.05 (0.041) [− 0.01, 0.14]	0.03 (0.03) [− 0.3, 0.11]	**0.04 (0.02)** [0.01, 0.9]	− 0.01 (0.02) [− 0.05, 0.02]	0.03 (0.02) [− 0.02, 0.08]	0.03 (0.03) [− 0.03, 0.08]
	*R*^*2*^	0.05	0.04	0.07	0.11	0.05	0.04
Appropriateness (IAM)							
	*b*	**0.18 (0.09)** [0.01, 0.40]	0.06 (0.07) [− 0.08, 0.21]	0.14 (0.08) [− 0.03, 0.30]	− 0.27 (0.16) [− 0.54, 0.07]	0.01 (0.08) [− 0.14, 0.18]	0.00 (0.09) [− 0.16, 0.21]
	*direct*	0.04 (0.10) [− 0.18, 0.23]	0.11 (0.09) [− 0.08, 0.26]	0.11 (0.09) [− 0.08, 0.27]	0.15 (0.08) [− 0.02, 0.30]	0.15 (0.10) [− 0.08, 0.31]	0.13 (0.09) [− 0.07, 0.30]
	*indirect*	**0.09 (0.05)** [0.01, 0.21]	0.03 (0.04) [− 0.04, 0.13]	0.04 (0.03) [− 0.01, 0.11]	− 0.01 (0.02) [− 0.07, 0.02]	0.02 (0.03) [− 0.05, 0.07]	0.00 (0.04) [− 0.07, 0.09]
	*R*^*2*^	0.07	0.05	0.07	0.10	0.05	0.04
Feasibility (FIM)							
	*b*	**0.16 (0.09)** [0.00, 0.35]	0.09 (0.07) [− 0.03, 0.22]	**0.17 (0.08)** [0.01, 0.31]	− 0.27 (0.15) [− 0.51, 0.06]	0.04 (0.07) [− 0.09, 0.19]	0.31 (0.07) [− 0.10, 0.18]
	*direct*	− 0.01 (0.10) [− 0.21, 0.19]	0.03 (0.09) [− 0.13, 0.20]	0.03 (0.09) [− 0.13, 0.21]	0.08 (0.09) [− 0.06, 0.03]	0.07 (0.09) [− 0.14, 0.24]	0.04 (0.09) [− 0.13, 0.22]
	*indirect*	**0.08 (0.05)** [0.00, 0.18]	0.05 (0.04) [− 0.02, 0.13]	**0.05 (0.03)** [0.02, 0.11]	− 0.01 (0.02) [− 0.08, 0.26]	0.01 (0.03) [− 0.03, 0.08]	0.05 (0.03) [− 0.05, 0.08]
	*R*^*2*^	0.07	0.06	0.08	0.11	0.06	0.05

Sample of therapists that received training in trauma screening tools (*N* = 494). Unstandardized parameters. Estimate (standard error) [95% confidence interval]. Independent variable is implementation leadership at T1 in all models. Global climate is a revised version of the ICS consisting of 14 items (excluding item 9 and the Rewards subscale). Recognition-r does not include item 9 from the original ICS. 95% bootstrapped confidence intervals (CIs) for all parameters. Parameters in bold have Cis that do not cross zero. LOCI cohort, age, gender, years of work experience in current occupation, and organizational tenure controlled for in all models

**Table 7:** Parameter estimates for direct and indirect effects (through implementation climate subscales) of implementation leadership on anticipated outcomes of trauma treatment methods at T3 (*N* = 184)

Outcome	Parameter	Mediator					
		Focus	Education support	Recognition-r	Rewards	Selection for EBP	Selection for openness
Implementation outcome							
	* a*	**0.43 (0.11)** [0.24, 0.68]	**0.47 (0.15)** [0.23, 0.79]	**0.26 (0.13)** [0.03, 0.51]	0.00 (0.07) [− 0.12, 0.19]	**0.29 (0.09)** [0.12, 0.50]	**0.43 (0.11)** [0.25, 0.66]
Acceptability (AIM)							
	* b*	**0.19 (0.09)** [0.02, 0.37]	0.11 (0.08) [− 0.04, 0.26]	**0.21 (0.07)** [0.04, 0.35]	− 0.04 (0.22) [− 0.44, 0.52]	0.06 (0.10) [− 0.12, 0.26]	0.03 (0.07) [− 0.11, 0.14]
	* direct*	0.11 (0.07) [− 0.02, 0.24]	**0.14 (0.07)** [0.02, 0.29]	**0.15 (0.05)** [0.05, 0.27]	**0.20 (0.05)** [0.09, 0.32]	**0.18 (0.07)** [0.05, 0.31]	**0.18 (0.06)** [0.07, 0.31]
	* indirect*	**0.08 (0.04)** [0.01, 0.18]	0.05 (0.04) [− 0.02, 0.13]	**0.05 (0.03)** [0.00, 0.12]	0.00 (0.02) [− 0.05, 0.03]	0.02 (0.03) [− 0.04, 0.08]	0.01 (0.03) [− 0.06, 0.06]
	* R*^*2*^	0.22	0.20	0.30	0.18	0.18	0.17
Appropriateness (IAM)							
	* b*	0.13 (0.14) [-0.11, 0.43]	0.12 (0.10) [− 0.09, 0.29]	**0.28 (0.09)** [0.09, 0.45]	− 0.17 (0.35) [− 0.75, 0.66]	0.07 (0.12) [− 0.18, 0.32]	0.02 (0.08) [− 0.17, 0.17]
	* direct*	0.17 (0.10) [− 0.04, 0.34]	**0.19 (0.08)** [0.00, 0.34]	**0.19 (0.07)** [0.04, 0.32]	**0.25 (0.08)** [0.08, 0.40]	**0.24 (0.09)** [0.07, 0.40]	**0.24 (0.08)** [0.09, 0.40]
	* indirect*	0.06 (0.07) [− 0.05, 0.21]	0.06 (0.05) [− 0.05, 0.15]	**0.07 (0.04)** [0.00, 0.17]	− 0.00 (0.03) [− 0.09, 0.04]	0.02 (0.04) [− 0.05, 0.10]	0.01 (0.04) [− 0.09, 0.07]
	* R*^*2*^	0.13	0.14	0.25	0.15	0.14	0.13
Feasibility (FIM)							
	* b*	0.17 (0.18) [− 0.18, 0.56]	0.24 (0.15) [− 0.07, 0.51]	**0.35 (0.12)** [0.10, 0.57]	− 0.04 (0.54) [− 0.71, 1.11]	0.12 (0.17) [− 0.21, 0.47]	0.05 (0.13) [− 0.25, 0.27]
	* direct*	0.20 (0.15) [− 0.11, 0.48]	0.18 (0.13) [− 0.07, 0.42]	**0.23 (0.09)** [0.05, 0.40]	**0.30 (0.09)** [0.11, 0.49]	**0.28 (0.10)** [0.08, 0.46]	**0.28 (0.10)** [0.10, 0.49]
	* indirect*	0.07 (0.09) [− 0.07, 0.27]	0.11 (0.09) [− 0.04, 0.29]	**0.09 (0.05)** [0.01, 0.21]	0.00 (0.03) [− 0.07, 0.06]	0.04 (0.05) [− 0.05, 0.14]	0.02 (0.06) [− 0.13, 0.12]
	* R*^*2*^	0.14	0.20	0.26	0.14	0.16	0.14

Sample of therapists that received training in trauma treatment methods (*N* = 101). Unstandardized parameters. Estimate (standard error) [95% confidence interval]. Independent variable is implementation leadership at T3 in all models. Global climate is a revised version of the ICS consisting of 14 items (excluding item 9 and the Rewards subscale). Recognition-r does not include item 9 from the original ICS. 95% bootstrapped confidence intervals (CIs) for all parameters. Parameters in bold have CIs that do not cross zero. LOCI cohort, age, gender, years of work experience in current occupation, and organizational tenure controlled for in all models

### Associations between implementation climate and EBP acceptability, appropriateness, and feasibility

The results indicated significant positive associations between implementation climate and acceptability and feasibility of the *screening tools* and acceptability, appropriateness, and feasibility of the *treatment methods* (Table 5, *b* parameters). In the model predicting appropriateness of the *screening tools*, the estimate was positive but non-significant. Thus, the results provided full support for hypothesis 3 that implementation climate assessed at T2 would be associated with EBP acceptability, appropriateness, and feasibility of *treatment methods* assessed 4 months later (T3) and partial support regarding the *screening tools.*

### Associations between specific aspects of perceived implementation climate and EBP acceptability, appropriateness, and feasibility

None of the specific aspects of perceived implementation climate (measured by subscales in the ICS) were consistently associated with the implementation outcomes (acceptability, appropriateness, and feasibility of screening tools and treatment methods) across all models. In models predicting perceptions of *screening tools* (Table 6, *b* parameters), associations were positive and significant between the Focus subscale and appropriateness and feasibility and between the Recognition-r subscale and acceptability and feasibility. The remaining estimates were non-significant.

In models predicting perceptions of *treatment methods* (Table 7, *b* parameters), the associations between the Focus subscale and acceptability were positive and significant, and Recognition-r was positively and significantly associated with all three outcomes. The remaining estimates were non-significant.

### Mediated associations between implementation leadership and EBP acceptability, appropriateness, and feasibility through implementation climate

The results indicated that perceived implementation climate significantly mediated parts of the associations between implementation leadership and the acceptability and feasibility of *screening tools* and those between implementation leadership and the acceptability, appropriateness, and feasibility of *treatment methods* (Table 5, *indirect* parameters). In the model predicting appropriateness of screening tools, the estimate was positive but non-significant. Thus, the results provided support for hypothesis 4 that implementation climate (T2) would mediate the associations between implementation leadership (T1) and therapists’ acceptability, appropriateness, and feasibility of the *treatment methods* (T3) and therapists’ acceptability and feasibility of the *screening tools.* The effect sizes and the explained variance (Table 5, R-squared) were higher for the *treatment methods* than the *screening tools*.

### Mediated associations between implementation leadership and EBP acceptability, appropriateness, and feasibility through specific aspects of perceived implementation climate

Regarding the *screening tools*, the results indicated that parts of the associations between implementation leaderships and outcomes were significantly mediated by the Focus subscale (appropriateness and feasibility) and the Recognition-r subscale (acceptability and feasibility) (Table 6, *indirect* parameters). Regarding the *treatment methods*, the results also indicated significant paths through the Focus subscale (acceptability) and the Recognition-r subscale (acceptability, appropriateness, and feasibility) (Table 7, *indirect* parameters). For the Recognition-r subscale, mediation was partial, with a significant *direct* path included. The results also indicated *direct* paths between implementation leadership and acceptability, appropriateness, and feasibility in the Educational support, Rewards, Selection for EBP, and Selection for openness models (Table 7, *direct* parameters). The Educational support, Rewards, Selection for EBP, and Selection for openness subscales did not significantly mediate associations between implementation leadership and any of the outcomes.

## Discussion

All four study hypotheses were fully confirmed for the treatment methods but only partially for the screening tools. Implementation leadership at T1 was significantly associated with therapists’ perceptions of acceptability, appropriateness, and feasibility of treatment methods, but not screening tools 8 months later (hypothesis 1). Implementation leadership at T1 was also significantly associated with perceived implementation climate 4 months later (hypothesis 2). Moreover, perceived implementation climate at T2 was associated with therapists’ perceptions of acceptability and feasibility (but not appropriateness) of the screening tools, and acceptability, appropriateness, and feasibility of the treatment methods 4 months later (hypothesis 3). Overall, implementation climate mediated the parts of the associations between implementation leadership and therapists’ perceptions of acceptability and feasibility (but not appropriateness) of screening tools, and acceptability, appropriateness, and feasibility of treatment methods (hypothesis 4).

The mediating role of perceived implementation climate between implementation leadership and various implementation outcomes supports previous findings implying leaders have a key role in the implementation process [15, 27] and that leaders can shape the implementation climate to support implementation of EBPs [45]. Although implementation climate mediated the association between implementation leadership and implementation outcomes of both screening tools and treatment methods, there was a tendency for the climate to matter more for therapists’ perceptions of the treatment methods than of the screening tools, both with regard to the larger effect sizes and explained variance.

Analyses with the implementation climate subscales showed a similar pattern with implementation leadership, such that associations were stronger for therapists’ perceptions of treatment methods than of screening tools. Only a subgroup of therapists was trained in the treatment methods, while the screening tools were implemented by all therapists. It might be that as screening is implemented by everyone, a therapist can more easily observe or seek guidance from a peer to inform their implementation, compared to being a smaller number of therapists implementing a more complex treatment method. Having fewer opportunities to observe or seek guidance from peers may require more support from the leaders. Thus, their leaders’ adaptation to target each therapist’s individual needs, both directly and through the implementation climate, might have been more decisive for the therapists’ perceived acceptability, appropriateness, and feasibility of the treatment methods than the screening tools.

Further exploration of which implementation climate factors influence implementation outcomes can give leaders a better understanding of what to emphasize during an EBP implementation. In this study, there are two implementation climate subscales associated with therapists’ reports of acceptability, appropriateness, and feasibility of treatment methods and screening tools, namely Focus and Recognition-r. All factors were measured during an active implementation phase [38], where leaders through their LOCI follow-up worked a lot on focusing on the methods and tools being implemented and recognizing the therapists’ achievements. This may have influenced the therapists’ perceptions of Focus and Recognition as being important in the active implementation phase and perhaps more than the other subscales. At the same time, it is surprising that subscales such as Educational support did not obtain significant results. The results are also inconsistent for each of the implementation outcomes, except the Recognition-r subscale on treatment methods. However, the absence of findings does not necessarily mean that there is no connection. It might be that different implementation climate factors are more important than others depending on different implementation phases, outcomes, or what is being implemented. This should therefore be further investigated.

The study supports the implementation leadership model [10], hypothesizing that leaders can achieve better implementation outcomes when they are supportive, knowledgeable, proactive, and perseverant when implementing specific EBPs.

This study includes a complex design with many clinics. Although the clinics were not randomized based on the current factors, time-lagged analyses make it possible to describe the precise sequence of operations through which an effect may occur [3]. However, we only measured each concept at one timepoint and consequently could not investigate possible changes in implementation leadership, implementation climate, and anticipated implementation outcomes over time. The concepts may also have been affected by variables not measured in the study, such as leaders’ commitment and engagement in activities that promote implementation [8], molar climate [28], or the complexity of the practice [27]. Thus, results should be interpreted with caution and does not permit any definitive claims about causal relationships.

The implementation leadership and implementation climate scales were measured at the individual level. As perceptions of acceptability, appropriateness, and feasibility can be considered as individual-level phenomena, it seems reasonable that the individual’s understanding of the perceived implementation climate is related to the individuals’ understanding of the screening tools and the treatment methods [20]. Some argue that implementation climate is a unit-level construct, measuring collective perceptions of the climate in an organization [21]. Thus, relationships involving implementation climate should, ideally, be measured and analyzed in a multilevel fashion to capture both within- and between-unit effects. However, in our study, such models did not converge. Aggregation indices for implementation climate were also below recommended thresholds for aggregating data [44]. Thus, this study focused on studying associations on the individual-level. For future research, similar analyses with aggregated data or multi-level structural equation models that estimate both within- and between-effects could be useful.

We also acknowledge that the Rewards subscale and item 9 from the Recognition subscale were excluded from the ICS global scale due to lack of cultural relevance in the Norwegian health system context [41]. Comparisons with other studies should therefore be made cautiously.

In this study, we have measured anticipated implementation outcomes in the form of therapists’ perceived EBP acceptability, appropriateness, and feasibility. These are assumed to predict actual implementation outcome [13]. However, more research should be done on the leaders’ impact on actual outcomes.

## Conclusion

Results from the study suggest that leaders can achieve better implementation outcomes both directly and through implementation climate. Implementation leadership and climate seemed to be of higher importance for the smaller group of therapists implementing more complex treatment methods, than for the whole group implementing somewhat simpler screening tools. More research is needed to understand which factors within the implementation climate affect different implementation outcomes, for whom, and under what conditions.

## Data Availability

The dataset used in the current study is available from the corresponding author on reasonable request.

## Abbreviations

EBP: Evidence-based practice
LOCI: Leadership and organizational change for implementation
PTSD: Posttraumatic stress disorder
